# Alpha-enolase in viral target cells suppresses the human immunodeficiency virus type 1 integration

**DOI:** 10.1186/s12977-020-00539-9

**Published:** 2020-09-11

**Authors:** Naoki Kishimoto, Kengo Yamamoto, Nozomi Iga, Chie Kirihara, Towa Abe, Nobutoki Takamune, Shogo Misumi

**Affiliations:** 1 Department of Environmental and Molecular Health Sciences, Faculty of Medical and Pharmaceutical Sciences, Kumamoto University, Kumamoto, 862-0973 Japan; 2 Kumamoto Innovative Development Organization, Kumamoto University, Kumamoto, 860-8555 Japan

**Keywords:** Human immunodeficiency virus type 1, Alpha-enolase, Reverse transcription, Integration

## Abstract

**Background:**

A protein exhibiting more than one biochemical function is termed a moonlighting protein. Glycolytic enzymes are typical moonlighting proteins, and these enzymes control the infection of various viruses. Previously, we reported that glyceraldehyde 3-phosphate dehydrogenase (GAPDH) and alpha-enolase (ENO1) are incorporated into human immunodeficiency virus type 1 (HIV-1) particles from viral producer cells and suppress viral reverse transcription independently each other. However, it remains unclear whether these proteins expressed in viral target cells affect the early phase of HIV-1 replication.

**Results:**

Here we show that the GAPDH expression level in viral target cells does not affect the early phase of HIV-1 replication, but ENO1 has a capacity to suppress viral integration in viral target cells. In contrast to GAPDH, suppression of ENO1 expression by RNA interference in the target cells increased viral infectivity, but had no effect on the expression levels of the HIV-1 receptors CD4, CCR5 and CXCR4 and on the level of HIV-1 entry. Quantitative analysis of HIV-1 reverse transcription products showed that the number of copies of the late products (R/*gag*) and two-long-terminal-repeat circular forms of viral cDNAs did not change but that of the integrated (Alu-*gag*) form increased. In contrast, overexpression of ENO1 in viral target cells decreased viral infectivity owing to the low viral integration efficiency. Results of subcellular fractionation experiments suggest that the HIV integration at the nucleus was negatively regulated by ENO1 localized in the nucleus. In addition, the overexpression of ENO1 in both viral producer cells and target cells most markedly suppressed the viral replication.

**Conclusions:**

These results indicate that ENO1 in the viral target cells prevents HIV-1 integration. Importantly, ENO1, but not GAPDH, has the bifunctional inhibitory activity against HIV-1 replication. The results provide and new insights into the function of ENO1 as a moonlighting protein in HIV-1 infection.

## Background

Human immunodeficiency virus type 1 (HIV-1) replication depends on not only viral proteins, but also host proteins. Several studies demonstrated that host proteins play a critical role in HIV-1 replication with positive or negative regulation. For example, CD4, CCR5 and/or CXCR4 are required in the viral entry step by binding with viral envelope proteins, and lens epithelium-derived growth factor (LEDGF/p75) is utilized as a cofactor of HIV-1 integrase during integration [1–8]. In contrast, host proteins, such as SAM domain and HD domain-containing protein 1 and apolipoprotein B mRNA-editing enzyme-catalytic polypeptide-like-3G, suppress HIV-1 replication in the early phase by degrading dNTPs and inducing G-to-A hypermutation in the viral genome, respectively [9–12]. Therefore, identification of the host proteins involved in HIV-1 replication is one way to understand their regulatory mechanism in HIV replication.

More than 300 proteins including glycolytic enzymes such as glyceraldehyde 3-phosphate dehydrogenase (GAPDH) and alpha-enolase (ENO1) have been identified as moonlighting proteins that perform more than one function [13–15]. Their multiple functions are not due to splicing variants, protein isoforms, or co-/post-translational modifications but to their diversity as receptors, scaffolds, enzymes, chaperones or transcription factors. Interestingly, GAPDH is an archetypal moonlighting protein that is involved in glycolysis, the carbon reduction cycle, the exportation of nuclear RNA, DNA repair and apoptosis [15–18]. Furthermore, GAPDH binds to viral RNA, such as the hepatitis A virus, hepatitis C virus and human parainfluenza virus, to regulate viral replication [19–21]. In addition, ENO1, which was initially identified as a glycolytic enzyme that catalyzes the conversion of 2-phosphoglycerate to phosphoenolpyruvate in glycolysis, is also a moonlighting protein functioning as a plasminogen receptor, a heat-shock protein and a hypoxic stress protein, and associates with infectious viruses such as the Sendai and dengue viruses [22, 23]. These studies raised the question of whether glycolytic enzymes can regulate HIV-1 replication as moonlighting proteins. We previously demonstrated that GAPDH binds to HIV-1 precursor proteins and is incorporated into virions to prevent the incorporation of the tRNA^Lys3^ complex and inhibits the initiation of HIV-1 reverse transcription [24, 25]. Moreover, we found that packaging of ENO1 into viral particles affects the early stage of viral reverse transcription [26]. However, the effects of the moonlighting functions of GAPDH and ENO1 on HIV-1 target cells have not been clarified.

In this study, we found that GAPDH expressed in HIV-1 target cells does not affect HIV-1 replication, but ENO1 expressed in HIV-1 target cells has the ability to decrease the efficiency of virus integration without altering the reverse transcription efficiency. Considering that ENO1 incorporated into virions suppresses the early stage of reverse transcription, it is reasonable to hypothesize that ENO1 has bifunctional inhibitory activity against HIV-1 replication. These findings provide a novel regulatory mechanism of HIV-1 replication by host proteins.

## Results

### ENO1 in HIV-1 target cells influences viral infectivity

**Fig. 1:**
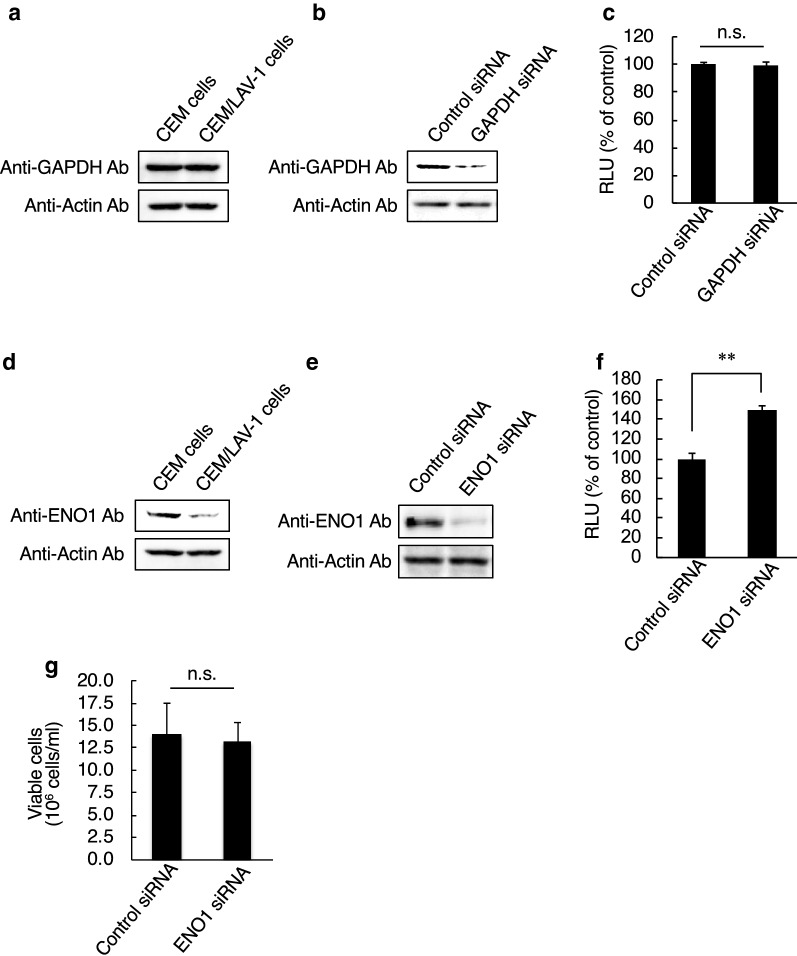
ENO1, but not GAPDH, in target cells affects HIV-1 replication. **a** Western immunoblotting monitoring of GAPDH expression in either CEM cells or CEM/LAV-1 cells. **b** Confirmation of knockdown efficiency of GAPDH-specific siRNA in TZM-bl cells. **c** HIV-1_LAV-1_ infectivity in either control or GAPDH-knockdown TZM-bl cells. The value in the control-siRNA-treated TZM-bl cells was set as 100%. **d** Western immunoblotting monitoring of ENO1 expression in either CEM cells or CEM/LAV-1 cells. **e** Confirmation of knockdown efficiency of ENO1-specific siRNA in TZM-bl cells. **f** HIV-1_LAV-1_ infectivity in ENO1-knockdown TZM-bl cells. The infectivity in the control-siRNA-treated TZM-bl cells was set as 100%. **g** Effects of ENO1 siRNA treatment on viability of TZM-bl cells. Viable cells were evaluated by trypan blue staining. The infectivity was examined at 2 days postinfection with HIV-1_LAV-1_ and assessed on the basis of the luciferase activity in lysates of any siRNA-treated TZM-bl cells. Data are mean values ± SE from triplicate tests. The significance of difference (Student’s *t-test*) is indicated as follows: **, *p* < 0.01; n.s., not significant

We previously reported that GAPDH and ENO1 are incorporated into HIV-1 particles, and virion-incorporated GAPDH and ENO1 inhibit viral reverse transcription independently of each other [24, 26]. Therefore, in this study, we examined whether GAPDH and ENO1 expression levels were changed by HIV-1 infection. Cell lysates derived from a T-cell line, CEM cells, and a chronically HIV-1-infected T-cell line, CEM/LAV-1 cells, were subjected to western immunoblotting. As a result, we detected no significant difference in the expression level of GAPDH between CEM and CEM/LAV-1 cells (Fig. 1a). In addition, GAPDH knockdown in TZM-bl cells maintained comparable levels of HIV-1 infectivity (Fig. 1b, c). On the other hand, the expression level of ENO1 was lower in CEM/LAV-1 cells than in CEM cells (Fig. 1d), and ENO1 knockdown in TZM-bl cells increased HIV-1 infectivity (Fig. 1e, f) without affecting cell viability (Fig. 1g). These findings suggest that ENO1 may have higher inhibitory activity against HIV-1 infection than GAPDH in target cells, and ENO1 in viral target cells may inhibit HIV-1 replication via a mechanism similar to or different from that underlying the inhibitory activity of virion-packaged ENO1.

### ENO1 knockdown in HIV-1 target cells increases viral integration efficiency

**Fig. 2:**
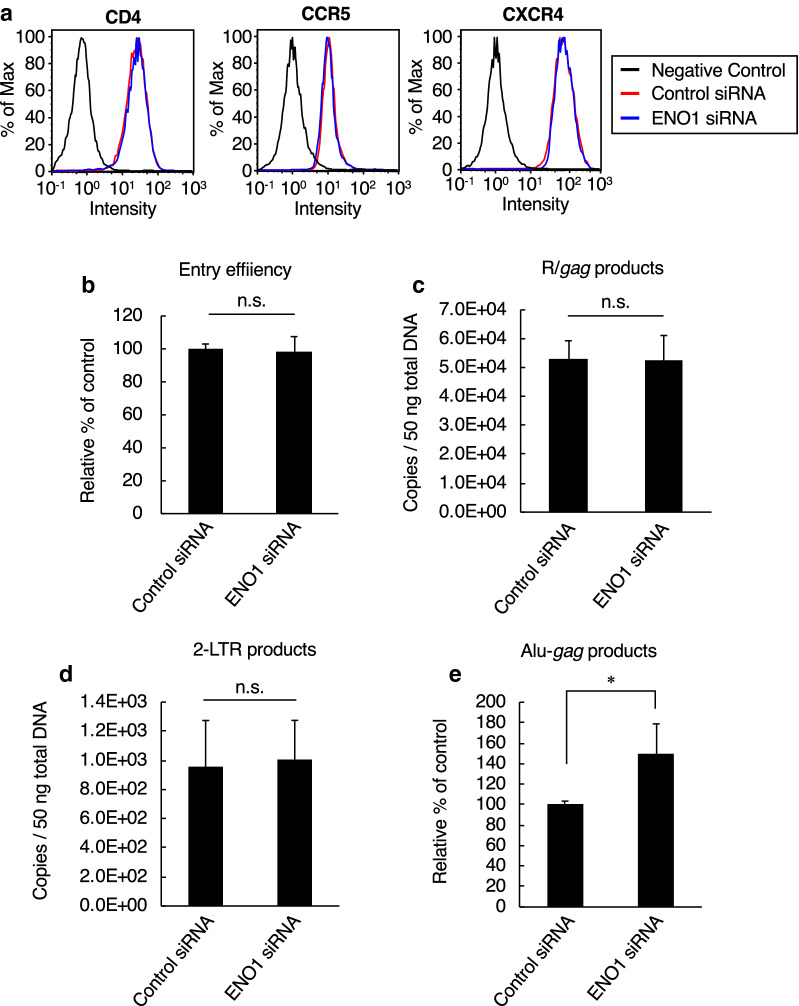
Effects of ENO1 knockdown in HIV-1 target cells on postentry step. **a** Flow cytometry monitoring of HIV-1 receptor CD4 and coreceptors CCR5 and CXCR4. Cell stained with isotype control antibody are indicated as negative control (black line). The expression levels of CD4, CCR5 and CXCR4 in control-siRNA (red line) or ENO1-specific-siRNA (blue line) -treated TZM-bl are shown. The expression level of each protein was examined just before the cells were infected with the virus. **b** Entry efficiency of HIV-1_LAV-1_ in either control or ENO1-knockdown TZM-bl cells. Entry efficiency was assessed on the basis of the amount of p24 from the cytosolic fraction of TZM-bl cells. The entry efficiency in the control-siRNA-treated TZM-bl cells was set as 100%. **c** Effect of ENO1 knockdown in TZM-bl cells on viral reverse transcription. The amount of R/*gag* products of viral reverse transcription was determined by quantitative real-time PCR analysis. **d** Effect of ENO1 knockdown in TZM-bl cells on viral cDNA nuclear import. The amount of 2-LTR circle products was determined by quantitative real-time PCR analysis. **e** Integration efficiency of HIV-1_LAV-1_ in either control or ENO1-knockdown TZM-bl cells. Relative amount of Alu-*gag* products was determined by nested-PCR. The integration efficiency in the control-siRNA-treated TZM-bl cells was set as 100%. Data are mean values ± SE from triplicate tests. The significance of difference (Student’s *t-test*) is indicated as follows: *, *p* < 0.05; n.s., not significant

To gain further insight into the function of ENO1 in HIV-1 target cells, we examined postentry steps in ENO1 knockdown cells. As shown in Fig. 2a, flow cytometry showed that treatment of TZM-bl cells with ENO1-specific siRNA had no effect on surface expression levels of the HIV-1 receptors CD4, CCR5 and CXCR4. In addition, measurement of cytosolic p24 isolated from HIV-1-infected TZM-bl cells by a previously described method [27] demonstrated that HIV-1 was able to penetrate into target cells regardless of the ENO1 expression level in the cells (Fig. 2b). We next investigated whether ENO1 knockdown in TZM-bl cells enhances the viral reverse transcription because low-level-ENO1-packaging virus, which was prepared by transfection of CEM/LAV-1 cells with an ENO1-specific siRNA, showed an increased reverse transcription efficiency [26]. Unexpectedly, ENO1 knockdown has no effect on the abundance of late R/*gag* products of reverse transcription (Fig. 2c). Furthermore, the number of copies of two-long-terminal-repeat (2-LTR) circular DNA products, which are generally used as a marker of viral cDNA nuclear import, also showed no significant difference between ENO1-knockdown cells and control cells (Fig. 2d). However, when we performed nested Alu-*gag* PCR analysis, which is generally used for calculation of integrated viral cDNA, we found that ENO1 knockdown increased the integration efficiency, which correlates with an enhanced HIV-1 infection (Fig. 2e). These findings indicate that unlike the inhibitory effect of virion-packaged ENO1, ENO1 in viral target cells inhibits HIV replication by preventing HIV-1 integration.

### ENO1 overexpression in HIV-1 target cells decreases viral integration efficiency

**Fig. 3:**
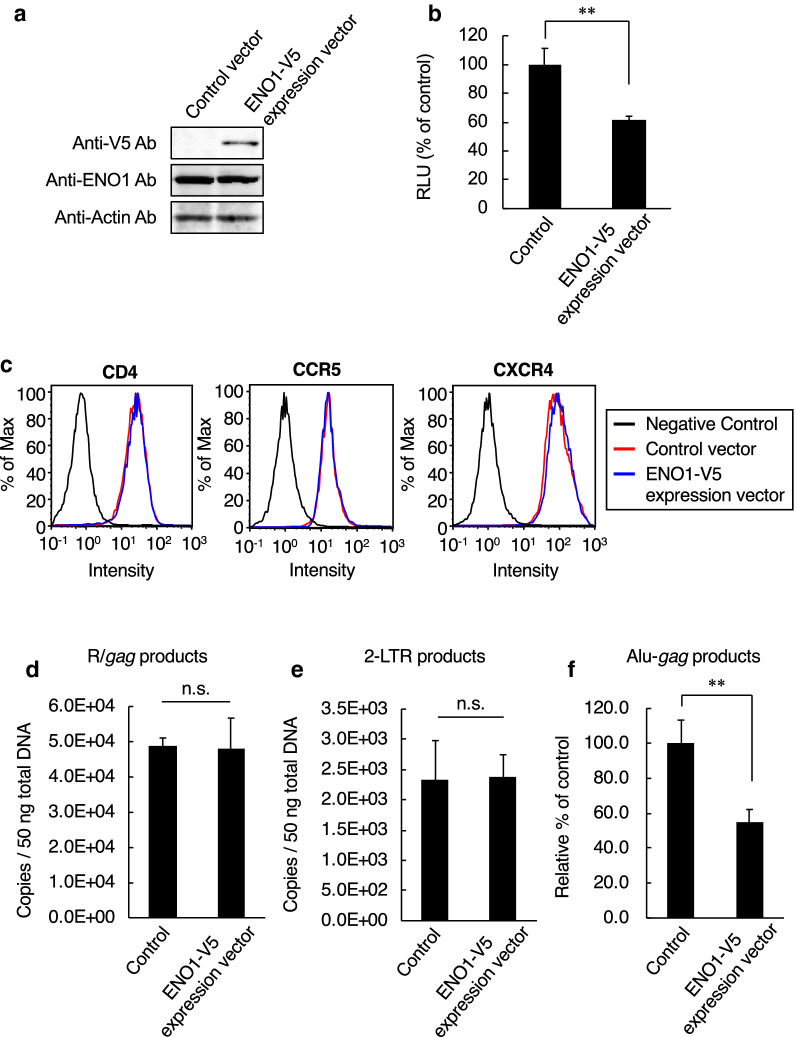
Effects of ENO1 overexpression in HIV-1 target cells on postentry step. **a** Confirmation of ENO1-V5 expression in TZM-bl cells. Endogenous ENO1 was detected with an anti-ENO1 antibody and exogenous ENO1 (ENO1-V5) was detected with an anti-V5 antibody. **b** HIV-1_LAV-1_ infectivity in ENO1-V5 expression vector-treated TZM-bl cells. The infectivity was examined at 2 days postinfection with HIV-1_LAV-1_ and assessed on the basis of the luciferase activity in lysates of vector-treated TZM-bl cells. The infectivity in the control-vector-treated TZM-bl cells was set as 100%. **c** Flow cytometry monitoring of HIV-1 receptor CD4 and coreceptors CCR5 and CXCR4. Cells stained with the isotype control antibody are indicated as negative control (black line). The expression levels of CD4, CCR5 and CXCR4 in control-vector- (red line) or ENO1-V5-expression vector (blue line)-treated TZM-bl cells are shown. Each protein was detected on the infection day. **d** Effect of ENO1-V5 expression in TZM-bl cells on viral reverse transcription. The amount of R/*gag* products of viral reverse transcription was determined by quantitative real-time PCR analysis. **e** Effect of ENO1-V5 expression in TZM-bl cells on viral cDNA nuclear import. The amount of 2-LTR circle products was determined by quantitative real-time PCR analysis. **f** Integration efficiency of HIV-1_LAV-1_ in either control- or ENO1-V5-expression vector-treated TZM-bl cells. Relative amount of Alu-*gag* products was determined by nested-PCR. The integration efficiency in the control-vector-treated TZM-bl cells was set as 100%. Data are mean values ± SE from triplicate tests. The significance of difference (Student’s *t-test*) is indicated as follows: **, *p* < 0.01; *, *p* < 0.05; n.s., not significant

Because ENO1 knockdown in the viral target cells resulted in increased HIV-1 infectivity, we next investigated the effect of ENO1 overexpression. To overexpress ENO1 in viral target cells, TZM-bl cells were transfected with an ENO1-V5 expression vector (Fig. 3a). As a result, ENO1 overexpression in HIV-1 target cell decreased viral integration efficiency (Fig. 3b). This result indicated that ENO1 impaired HIV-1 infection in viral target cells. To further examine whether ENO1 overexpression affects CD4, CXCR4 and CCR5 expression levels, we performed flow cytometry using anti-CD4, anti-CCR5 and anti-CXCR4 antibodies. The results showed that ENO1 overexpression had no effect on the expression levels of HIV-1 receptors, indicating that the viral entry step was unaffected by ENO1 overexpression (Fig. 3c). In addition, quantitative real-time PCR showed that ENO1 overexpression also had no effect on the number of copies of late R/*gag* (Fig. 3d) and 2-LTR circular DNA products (Fig. 3e). However, as expected, nested Alu-*gag* PCR analysis showed that ENO1 overexpression decreased viral integration efficiency compared with control vector treatment (Fig. 3f). Previously, we reported that high-level-ENO1-packaging virus, which was prepared by cotransfection of HEK293 cells with pNL-CH and ENO1-V5 expression vector, showed decreased number of copies of viral reverse transcription products [26]. Therefore, on the basis of these findings, we hypothesized that ENO1 is a bifunctional inhibitory protein that inhibits reverse transcription and integration processes.

### Nuclear ENO1 prevents HIV-1 integration

**Fig. 4:**
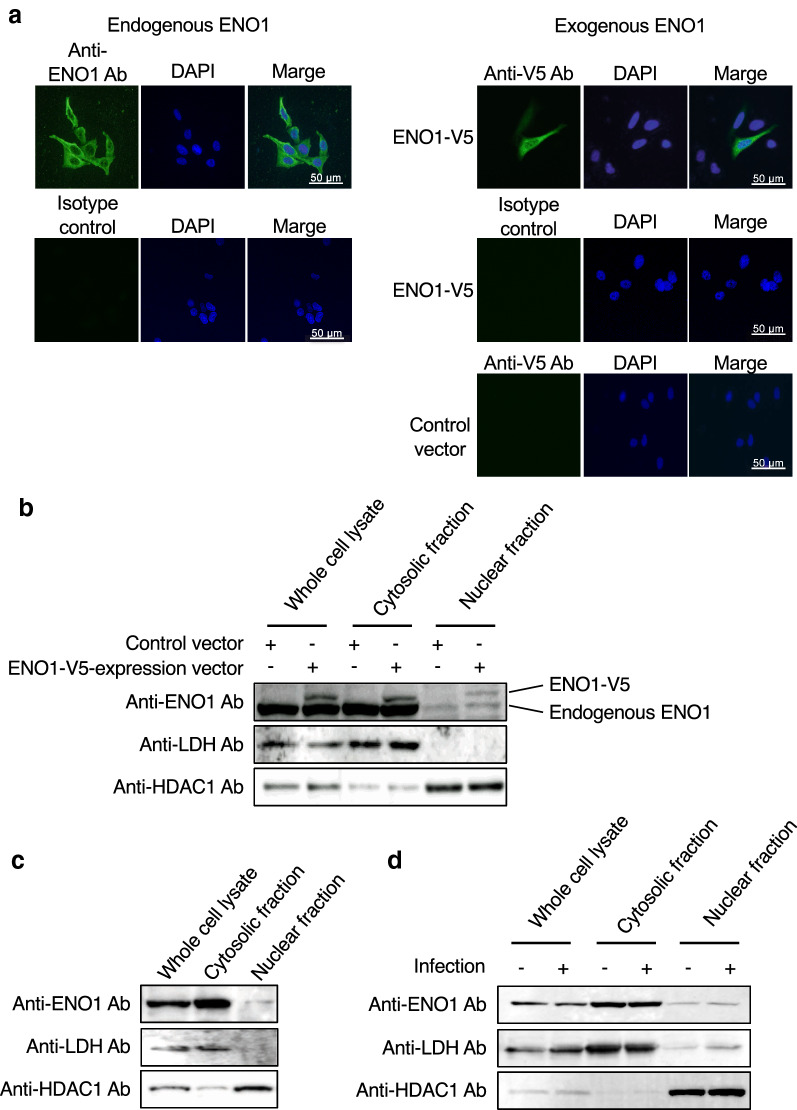
ENO1 is present in the nucleus of the target cell. **a** Observation of endogenous ENO1 and ENO1-V5 by fluorescence microscopy. Endogenous ENO1 (left top panel) and ENO1-V5 (right top panel) were visualized by staining with the anti-ENO1 antibody and anti-V5 antibody, respectively. Nuclei were visualized by DAPI staining (*middle column*). The ENO1 or ENO1-V5 signal and DAPI signal are shown as merged (*right column*). In the right lower panels, cells transfected with the control vector were also analyzed. Small amounts of ENO1 and ENO1-V5 colocalized in the nucleus. A representative image from three independent experiments is shown. Subcellular fractionation of **b** TZM-bl cells, **c** CEM cells and **d** HIV-1_LAV-1_-infected TZM-bl cells. **b**–**d** Endogenous ENO1 was detected by staining with the anti-ENO1 antibody and ENO1-V5 was detected by staining with the anti-V5 antibody. LDH and HDAC1 were detected as the cytosolic and nuclear markers, respectively. Fractionation was performed from the same cell number and then the amount loaded to each lane was calculated by the BCA method

To prevent HIV-1 integration, ENO1 should be in the viral target cell nucleus. Therefore, we confirmed the subcellular localization of ENO1. Endogenous ENO1 in TZM-bl cells was stained with specific antibodies and detected by fluorescence microscopy. We observed a weak nuclear ENO1-specific signal (Fig. 4a, left top panel). Interestingly, a clearer signal from V5-tagged ENO1, which was expressed by transfection in TZM-bl cells, indicated that ENO1 was present in the nucleus (Fig. 4a, right top panel). To clarify the ENO1 localization in more detail, we next fractionated the cells and detected endogenous ENO1, ENO1-V5, lactate dehydrogenase (LDH) and histone deacetylase1 (HDAC1) by western immunoblotting. LDH and HDAC1 were detected as a cytosolic marker and a nuclear marker, respectively. As shown in Fig. 4b, large amounts of ENO1 and ENO1-V5 were detected in the cytosolic fraction. Interestingly, small amounts of ENO1 and ENO1-V5 were also detected in the nuclear fraction, suggesting that the suppression of HIV infection by ENO1 overexpression (Fig. 3f) depended on V5-tagged ENO1 translocated into the nucleus. To eliminate the effects of the V5-tag, treatment of the untagged ENO1 expression vector increased the amount of ENO1 in the nucleus (Additional file 1: Figure S1A) and enhanced the inhibitory effect of ENO1 (Additional file 1: Figure S1B). Next, since TZM-bl cells are derived from HeLa cells, not immune cells, we also determined whether a small amount of ENO1 is present in the nucleus of immune cells. CD4^+^ T-cell line CEM cells were fractionated into the cytosol and nucleus fractions using the same method as that for TZM-bl fractionation. As shown in Fig. 4c, a small amount of ENO1 located in the nucleus. To further confirm whether acute HIV-1 infection affects ENO1 nuclear translocation, TZM-bl cells were infected with HIV-1 and fractionated. As a result, the same amount of ENO1 was detected in the nuclear fraction from noninfected or infected cells (Fig. 4d), indicating that ENO1 nuclear localization was unaffected by acute HIV-1 infection. These results suggest that the larger the amount of ENO1 present in the nucleus, the more HIV-1 integration is inhibited.

### ENO1 has bifunctional inhibitory activities on HIV-1 infection

**Fig. 5:**
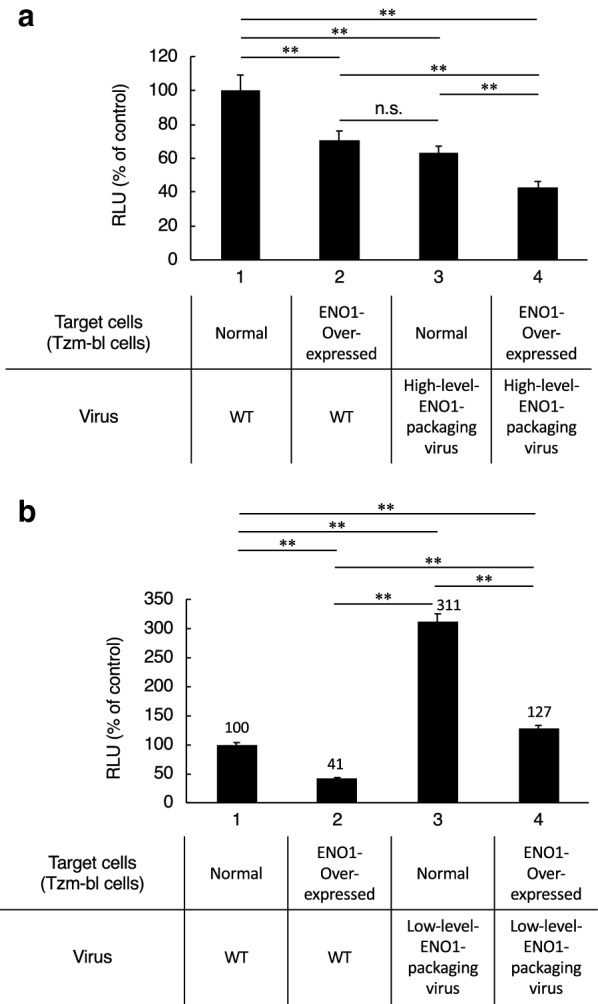
ENO1 in viral producer and viral target cells has bifunctional inhibitory activities against HIV-1 replication. **a** Infection assay to confirm bifunctional inhibitory activity of ENO1. A high-level-ENO1-packaging virus derived from pNL-CH-transfected HEK293 cells and ENO1-overexpressing TZM-bl cells were prepared with an ENO1-V5 expression vector. The bifunctional inhibitory activity of ENO1 was examined by infecting target cells with different ENO1 expression levels using viruses with different amounts of ENO1 incorporated into their particles. **b** Infection assay to confirm bifunctional inhibitory activity of ENO1. A low-level-ENO1-packaging virus derived from CEM/LAV-1 cells treated with ENO1-specific siRNA and ENO1-overexpressing TZM-bl cells were prepared. The value in the control experiment (target cells and producer cells are shown as normal or WT) was set as 100%. Data are mean values ± SE from triplicate tests. The significance of difference (Nonrepeated measures ANOVA and Dunnett’s test versus control) is indicated as follows: **, *p* < 0.01; n.s., not significant

Finally, we clarified the bifunctional inhibitory activities of ENO1 in more detail. First, we prepared a high-level-ENO1-packaging virus by cotransfection of HEK293 cells with pNL-CH and ENO1-V5 expression vector. As shown in Fig. 5a, lane 3, the high-level-ENO1-packaging virus showed significant decreases in its infectivity in normal TZM-bl cells. This finding is consistent with our previous findings [26]. Furthermore, as shown in Fig. 3b, the control virus showed significant decreases in its infectivity in the ENO1-overexpressing TZM-bl cells (Fig. 5a, lane 2). As expected, the high-level-ENO1-packaging virus showed a greater reduction in its infectivity in ENO1-overexpressing TZM-bl cells (Fig. 5a, lane 4). Second, we prepared a low-level-ENO1-packaging virus from culture supernatants of ENO1-specific-siRNA-treated CEM/LAV-1 cells. As shown in Fig. 5b, lanes 1 and 2, the control WT virus showed about 60% reduction in its infectivity in ENO1-overexpressing TZM-bl cells. In contrast, the low-level-ENO1-packaging virus also showed about 60% reduction in infectivity in the ENO1-overexpressing TZM-bl cells (Fig. 5b, lanes 3 and 4). These findings indicate that ENO1 in viral producer and target cells has bifunctional inhibitory activities on HIV-1 replication.

## Discussion

Glycolytic enzymes have diverse functions as moonlighting proteins, and also regulate various infectious viruses. For example, GAPDH regulates hepatitis A virus, hepatitis C virus and human parainfluenza virus infection and ENO1 regulates Sendai virus and dengue virus infection [19–23]. In addition, we previously focused on virion-associated GAPDH and ENO1, and found that they independently prevent HIV-1 replication in HIV-1 producer cells [24–26]. Here, we examined whether GAPDH and ENO1 in viral target cells are involved in HIV-1 replication and found that GAPDH in the viral target cells is not involved in HIV-1 replication, whereas ENO1 in the viral target cells prevents HIV-1 replication by a mechanism different from that of ENO1 in viral producer cells. This is the first result showing that ENO1 inhibits viral replication even in HIV target cells.

It has not been clarified whether ENO1 in viral target cells is involved in HIV-1 replication. Here we showed that ENO1 in viral target cells has the capacity to prevent HIV-1 integration. We previously demonstrated that ENO1 was packaged into HIV-1 particles and the packaging of ENO1 suppresses the early stage of viral reverse transcription without inhibiting the packaging of cellular tRNA^Lys3^ into viral particles [26]. Unexpectedly, we found that the overexpression of ENO1 in viral target cells did not affect the number of copies of early and late cDNA products of HIV-1 reverse transcription (Supplementary Fig. 2 in [26] and Fig. 3d). However, we found that nuclear ENO1 expression in the viral target cells prevented HIV-1 integration. The ENO1 gene can give rise to a short 37 kDa nuclear isoform, also called myc promoter binding protein 1 (MBP-1), which suppresses the activity of the c-myc transcription factor in the nucleus and lacks the first 96 amino acids [28]. In this study, as shown in Fig. 4b, we found that the same long form of ENO1 as the 48 kDa enolase present in the cytoplasmic fraction was translocated to the nucleus, but no short MBP-1 was observed. The presence of the nuclear localization signal of ENO1 has not been described for any human, parasite, or plant ENO1 that is efficiently transported to the nucleus for repression of gene transcription [28–30]. Mouveaux et al. demonstrated that ENO1 of *Toxoplasma gondii* (TgENO1) exclusively localized in the nucleus [31]. Since the expression of about 48 kDa HA-tagged TgENO1 has been confirmed by western immunoblotting [31], it is expected that its *N*-terminal long form has migrated into the nucleus and has the capacity for binding to putative gene promoters to control gene expression during the intracellular proliferation of *T. gondii* [31]. Furthermore, Cho et al. showed that ENOblock, a chemical probe for elucidating the moonlighting functions of ENO1, induces the nuclear localization of the long form of 48 kDa ENO1 [32]. It is generally considered that proteins with a molecular weight of about 40 kDa or larger cannot pass through the nuclear pore for their shuttling from the cytoplasm into the nucleoplasm. Interestingly, Cho et al. demonstrated that ENOblock enhances the nuclear localization of ENO1, but the effect of ENOblock is suppressed by treatment with an O-GlcNAc transferase inhibitor, OSMI-1 [33]. Although 10 μM ENOblock did not promote ENO1 nuclear translocation in TZM-bl cells, compounds with the same action as ENOblock may suppress HIV integration by promoting 48 kDa ENO1 translocation into the nucleus.

In the integration process, the viral integrase forms a preintegration complex (PIC) with a reverse-transcribed viral cDNA and cellular essential cofactors such as LEDGF/p75 and cleavage and polyadenylation specificity factor subunit 6 (CPSF6) [6–8, 34]. Nuclear entry of HIV-1 PIC through the nuclear pore complex is an essential step in establishing HIV-1 infection. Bejarano et al*.* demonstrated that HIV-1 nuclear import in macrophages is regulated by CPSF6-capsid interactions at the nuclear pore complex [35]. Although there are differences between macrophages and cell lines, the expression level of ENO1 in the viral target cells did not affect the number of copies of 2-LTR circles forms (Figs. 2d, 3e), suggesting that ENO1 is not directly involved in the process of HIV-1 PIC nuclear translocation. Following nuclear import, HIV-1 PIC utilizes both CPSF6 and LEDGF/p75 to target integration into transcriptionally active genes [34–38]. Recently, Sowd et al*.* found that CPSF6 directs HIV-1 to transcriptionally active chromatin, where LEDGF/p75 predominantly directs the positions of integration within active genes [34]. To clarify how ENO1 prevents viral integration, we carried out a coimmunoprecipitation assay. As far as we investigated, the coimmunoprecipitation assay showed that ENO1 did not directly interact with HIV-1 integrase or LEDGF/p75 (data not shown). In addition, the yeast-two hybrid analysis demonstrated that ENO1 also did not interact with HIV-1 proteins such as capsid, reverse transcriptase and integrase (Supplementary Fig. 2 in [26]). Furthermore, an increased ENO1 expression level decreases the efficiency of viral integration, and a decreased ENO1 expression level increases it (Figs. 2e, 3f). These findings suggest that ENO1 does not inhibit viral integration by directly binding to integrase or LEDGF/p75 and may suppress the viral integration within active genes by its direct binding to nearby active genes, as demonstrated by Subramanian and Miller [28] that the amino acids between 96 and 236 amino acids of ENO1 are essential for DNA interaction.

Since viruses replicate in host cells, information about the cellular environment, such as metabolism, is important for understanding virus replication. Hegedus et al*.* reported that HIV-1 infection increases in glycolytic flux [39]. Glucose transporter 1 is used as the main glucose transporter in activated T-cells, and its surface expression level is upregulated in HIV-1-infected CD4^+^ T cells [40]. Furthermore, HIV-1 infection increases hexokinase expression level and activity [41]. These lines of evidence demonstrated that HIV-1 replication requires glycolysis-dependent cellular conditions. Interestingly, Hegedus et al*.* demonstrated that HIV-1 produced from cells cultured in galactose-containing medium, in which energy biosynthesis depends on oxidative phosphorylation and not glycolysis, showed decreased infectivity [39]. These findings led us to hypothesize that if glycolytic flux increases due to HIV-1 infection, glycolytic enzymes such as ENO1 must be involved in glycolysis, and HIV-1 replication will not be inhibited by ENO1.

## Conclusions

We conclude that ENO1 in viral target cells inhibits HIV-1 integration. Together with our previous report, we consider ENO1 has bifunctional inhibitory activity against HIV-1 replication. This study strongly supports the possibility that glycolytic enzymes act as moonlighting proteins to inhibit HIV-1 replication.

## Methods

### Cell culture

CEM cells of the a CD4^+^ T cell line and CEM/LAV-1 cells of the chronically HIV-1_LAV-1_-infected T-cell line were maintained at 37 °C in RPMI-1640 supplemented with 10% fetal calf serum containing 100 IU/ml penicillin and 100 μg/ml streptomycin in 5% CO_2_. TZM-bl cells and HEK293 cells were maintained in DMEM instead of RPMI-1640. TZM-bl cells were obtained from the NIH AIDS Research and Reference Reagent Program.

### Transfection

Cells were transfected with 100 nM validated commercially available Silencer™ GAPDH siRNA (Catalog #:AM4605, Thermo Fisher Scientific Inc.) to suppress GAPDH expression and with 100 nM validated commercially available Stealth ENO1 siRNA (Catalog #:HSS103243, Thermo Fisher Scientific Inc.) to suppress ENO1 expression using the Neon™ transfection system (Thermo Fisher Scientific Inc.). To overexpress ENO1, cells were transfected with previously prepared pcDNA™3.1D-ENO1-V5-His-TOPO^®^ (Thermo Fisher Scientific Inc.; ENO1-V5 expression vector) using Lipofectamine^®^ LTX reagent and Plus™ Reagent (Thermo Fisher Scientific Inc.). To prepare the untagged ENO1 expression vector, the coding region of human ENO1 was cloned into pEBMulti-Neo (Wako Pure Chemical Industries, Ltd.). All experiments were performed in accordance with manufacturer’s instructions. The expression level of each protein was determined by western immunoblotting using anti-GAPDH antibody (Merck KGaA), anti-ENO1 antibody (Santa Cruz Biotechnology, Inc.), anti-V5 antibody (Thermo Fisher Scientific Inc.), anti-actin antibody (Wako Pure Chemical industries, Ltd.) or HIV-1-positive plasma (a gift from Dr. Matsushita, Kumamoto University). The cytotoxicity induced by siRNA treatment was evaluated by trypan blue dye exclusion assay [24].

### Viruses

Infectious HIV-1_LAV-1_ stocks were prepared from culture supernatants of CEM/LAV-1 cells. The low-level-ENO1-packaging virus stocks were prepared from culture supernatants of ENO1-specific siRNA-treated CEM/LAV-1 cells, as previously described [26]. On the other hand, the high-level-ENO1-packaging virus stocks were prepared by cotransfection of HEK293 cells with the pNL-CH and ENO1-V5 expression vector, as previously described [26]. The release of each virus was directly monitored by p24 ELISA (ZeptoMetrix Corporation) as the amount of the CA protein in cell culture supernatants.

### Assessment of entry and postentry events

The expressions of CD4, CCR5 and CXCR4 at the cell surface were confirmed at 1 day posttransfection by flow cytometry analysis using anti-CD4 antibody (Biolegend Inc.), anti-CCR5 antibody and anti-CXCR4 antibody (R&D systems), respectively. The viral entry efficiency was determined by p24 ELISA (ZeptoMetrix Corporation) as the amount of the CA protein in the cytosolic fraction, as previously described [27]. Briefly, viral target cells were infected with each virus at 4 °C for 30 min, and then incubated further for 4 h at 37 °C. The cells were treated with 0.25% trypsin and then the cell cytosolic fraction was collected by two-step centrifugation (1st; 1000×*g*, 2nd; 195,480×*g*) after homogenization in swelling buffer [10 mM Tri-HCl (pH8.0), 10 mM KCl, 1 mM EDTA]. De novo synthesized viral cDNA products were measured by quantitative real-time PCR analysis in accordance with a previous methods [24, 26, 42]. To measure the amounts of R/*gag*, 2-LTR circle and Alu-*gag* products, DNA extracted from infected TZM-bl cells was amplified using the following primers. R/*gag* products: M667 (5′-GGCTAACTAGGGAACCCACTG-3′) and M661 (5′-CCTGCGTCGAGAGAGCTCCTCTGG-3′); 2-LTR circle products: 2-LTR sense (5′-GAGATCCCTCAGACCCTTTTAG-3′) and 2-LTR antisense (5′-GTCAGTCG- ATATCTGATCCCTG-3′); Alu-*gag* products: Alu-specific primer (5′-TCCCAGCTACTCGGGAGGCT- GAGG-3′) and M661. To determine viral integration efficiency, Alu-*gag* products were further amplified using M667 and AA55 (5′-CTGCTAGAGATTTTCCACACT- GAC-3′). Infectivity was determined by measuring the luciferase activity in cell lysates, which were prepared by a previously described method [20]. Viral infection was performed 24 h after transfection.

### Fluorescence microscopy and cell fractionation

TZM-bl cells were seeded on an 8-well chambered Nunc™ Lab-Tek™ II Chamber Slide™ system. At 24 h posttransfection, the cells were fixed with 1% paraformaldehyde, as previously described [43]. After that, endogenous ENO1 was detected with an anti-ENO1 antibody (Santa Cruz Biotechnology, Inc.) and exogenous ENO1 (ENO1-V5) was detected with an anti-V5 ENO1 antibody (Thermo Fisher Scientific Inc.). Normal Goat IgG control (Wako Pure Chemical industries, Ltd.) and mouse IgG2a (isotype control) (MEDICAL & BIOLOGICAL LABORATORIES CO., LTD.) were used as negative control. Cells were fractionated using Subcellular Protein Fractionation Kit for Cultured Cells (Thermo Fisher Scientific Inc.) in accordance with the manufacturer’s instructions. To fractionate cells after infection, the cells were seeded on a 6-well plate and incubated with HIV-1_LAV-1_ and 20 µg/ml DEAE dextran for 2 h and then cultured with cell growth medium for 48 h.

## Supplementary information

**Additional file 1: Figure S1.** Effects of untagged ENO1 in HIV-1 target cells.

## Data Availability

All data generated or analyzed during this study are included in this published article.

## Abbreviations

GAPDH: Glyceraldehyde 3-phosphate dehydrogenase
ENO1: Alpha-enolase
HIV-1: Human immunodeficiency virus type 1
LEDGF: Lens epithelium-derived growth factor
2-LTR: Two-long terminal repeat
LDH: Lactate dehydrogenase
HDAC1: Histone deacetylase1
MBP-1: Myc promoter binding protein 1
TgENO1: ENO1 of *Toxoplasma gondii*
PIC: Preintegration complex
CPSF6: Cleavage and polyadenylation specificity factor subunit 6
