# Supplementary material for: Alpha-enolase in viral target cells suppresses the human immunodeficiency virus type 1 integration
Source: Retrovirology. 2020 Sep 11;17:31. doi: 10.1186/s12977-020-00539-9 (PMC7488571; doi:10.1186/s12977-020-00539-9)
Supplement: Supplementary file 1 — Additional file 1: Figure S1. Effects of untagged ENO1 in HIV-1 target cells. [file 12977_2020_539_MOESM1_ESM.pdf]

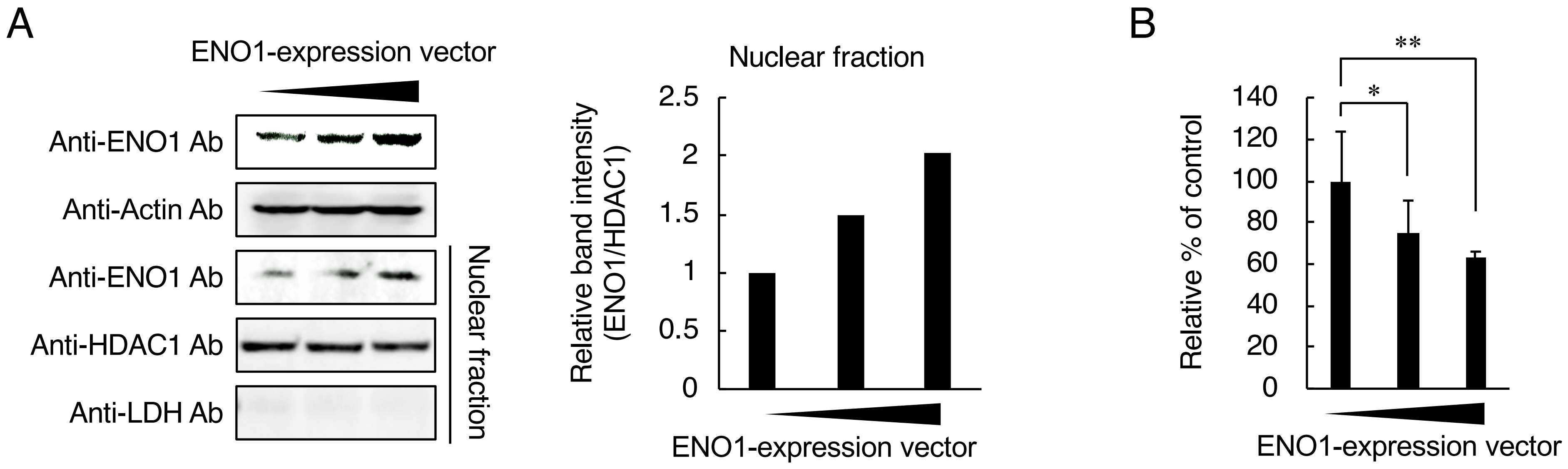

Figure S1. Effects of untagged ENO1 in HIV-1 target cells. (A) Untagged ENO1 expression and localization in TZM-bl cells. The left panel shows the results of western immunoblotting. The right panel shows the results of band intensity analysis. These results indicate that the amount of ENO1 localized in the nucleus increases in a transfected-DNA-amount-dependent manner. The nuclear fraction did not contain LDH (left panel, bottom). (B) Infection assay to confirm the effect of untagged ENO1 transfection. The significance of difference (Nonrepeated measures ANOVA and Dunnett's test versus control) is indicated as follows: **, $p<0.01$; *, $p<0.05$.
